# In Situ Synthesis of Highly Fluorescent, Phosphorus-Doping Carbon-Dot-Functionalized, Dendritic Silica Nanoparticles Applied for Multi-Component Lateral Flow Immunoassay

**DOI:** 10.3390/s24010019

**Published:** 2023-12-19

**Authors:** Jia-Xuan Hu, Shou-Nian Ding

**Affiliations:** Jiangsu Province Hi-Tech Key Laboratory for Bio-Medical Research, School of Chemistry and Chemical Engineering, Southeast University, Nanjing 211189, China; 220213216@seu.edu.cn

**Keywords:** carbon dots, *in situ* synthesis, multi-component detection, lateral flow immunoassay

## Abstract

The sensitivity of fluorescent lateral flow immunoassay (LFIA) test strips is compromised by the low fluorescence intensity of the signaling molecules. In this study, we synthesized novel phosphorus-doped carbon-dot-based dendritic mesoporous silica nanoparticles (DMSNs-BCDs) with a quantum yield as high as 93.7% to break this bottleneck. Meanwhile, the *in situ* growth method increased the loading capacity of carbon dots on dendritic mesoporous silica, effectively enhancing the fluorescence intensity of the composite nanospheres. Applied DMSNs-BCDs in LFIA can not only semi-quantitatively detect a single component in a short time frame (procalcitonin (PCT), within 15 min) but also detect the dual components with a low limit of detection (LOD) (carbohydrate antigen 199 (CA199) LOD: 1 U/mL; alpha-fetoprotein (AFP) LOD: 0.01 ng/mL). And the LOD of PCT detection (0.01 ng/mL) is lower by 1.7 orders of magnitude compared to conventional colloidal gold strips. For CA199, the LOD is reduced by a factor of four compared to LFIA using gold nanoparticles as substrates, and for AFP, the LOD is lowered by two orders of magnitude compared to colloidal gold LFIA. Furthermore, the coefficients of variation (CV) for intra-assay and inter-assay measurements are both less than 11%.

## 1. Introduction

The early and accurate detection of biomarkers is crucial for the diagnosis and monitoring of various diseases, including bacterial inflammatory diseases and tumors [1,2,3]. However, traditional detection methods such as enzyme-linked immunosorbent assay (ELISA) [4] often require longer detection times and higher costs, the electrochemical method [5,6,7,8] may lack portability, and chemiluminescence immunoassay [9,10,11] has a short luminescence process and poor stability. Lateral flow immunoassay (LFIA), as a widely used point-of-care testing (POCT) diagnostic tool, offers the advantages of rapidity, portability, low cost, and good stability [12,13,14,15]. Traditional LFIA uses gold nanoparticles to label Ab_2_, but due to significant batch-to-batch variations in colloidal gold products, the physical adsorption method used results in the easy detachment of antigens/antibodies from the gold particle surface, leading to marker instability and consequently lower detection sensitivity [16,17,18,19]. Therefore, it is imperative to develop novel materials to enhance detection sensitivity.

At present, the utilization of high-quantum-yield fluorescent nanomaterials as luminous signal substances has shown promising potential in fluorescence-based LFIA [20]. These nanomaterials have unique optical properties such as high brightness, photostability, and tunable emission wavelengths [21]. Carbon-dot-based fluorescent nanomaterials, known for their biocompatibility and high brightness, have garnered significant attention as suitable fluorescent markers for LFIA [22,23,24,25]. Carbon dots (CDs) are small carbon nanoparticles that are smaller than 10 nm and exhibit strong fluorescence [26,27]. Heteroatom doping can introduce new energy levels and improve the electronic structure of carbon dots, which contributes to enhancing their fluorescence performance. This makes them a better choice as a fluorescent label or probe for sensitive detection in biomedical and biochemical applications [28,29,30,31]. Gong et al. used hydro-heat treatment to carbonize adenosine-5′-triphosphate to obtain phosphorus and nitrogen-doped highly fluorescent carbon dots (PN-CDs), which can be used as sensitive sensors for the rapid imaging of reactive oxygen species (ROS) and reactive nitrogen species (RNS) signals with high selectivity and contrast [30]. Huang et al. synthesized fluorescent nitrogen and phosphorus double-doped CDs (N, P-CDs), using sucrose as a carbon source and 1,2-ethylenediamine (EDA) and phosphoric acid as dopants, and applied N, P-CD-based fluorescent probes to hemoglobin with a detection limit of 0.29 nM [32].

However, due to the complex surface functional groups of carbon dots, a carrier is required for their immobilization to facilitate their application in LFIA. Dendritic mesoporous silica nanoparticles (DMSNs) have gained considerable application prospects in the biomedical domain owing to their expansive radial pore structure and superior accessible surface area in comparison to compact silica nanoparticles [33]. Utilizing DMSNs as carriers not only promotes the stable luminescence of CDs but also amplifies the signal. Previous studies have demonstrated the compatibility of DMSNs with LFIA [12,34]. Gao et al. integrated quantum dots (QDs) into DMSNs for signal amplification in labeling materials, enabling the sensitive detection of C-reactive protein (CRP) with a detection limit of 5 pg/mL [35]. Huang et al. assembled hydrophobic quantum dots (QDs) with DMSNs, forming a pitaya-shaped fluorescent mesoporous silica colloid, which provides highly sensitive, specific, and robust immunoassays for CRP in clinical samples [36]. 

**Figure 1:**
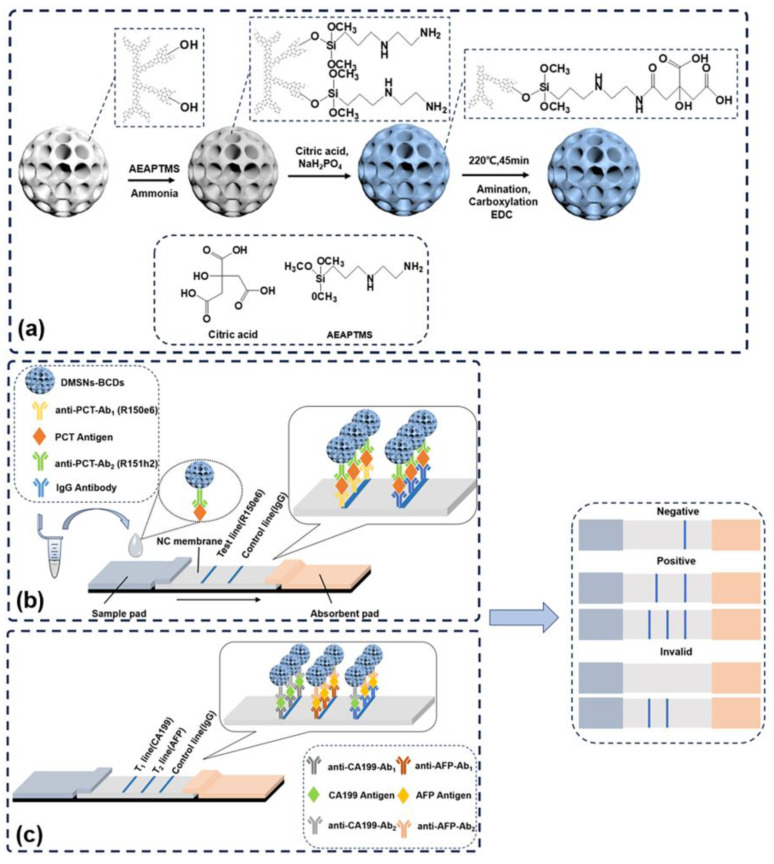
(**a**) Synthesis process diagram of DMSNs-BCDs; (**b**) the detection process of PCT using DMSNs-BCDs; (**c**) the detection process of CA199 and AFP using DMSNs-BCDs.

Herein, we synthesized novel, phosphorus-doped, carbon-dot-based DMSNs (DMSNs-BCDs) using an *in situ* growth method and utilized it to develop LFIA (DMSNs-BCDs-LFIA) for the single-component detection of PCT and the dual-component detection of CA199 and AFP (Figure 1). Phosphorus doping, the *in situ* growth of carbon dots, and the large specific surface area of DMSNs result in a high quantum yield (~93.7%) of DMSNs-BCDs [37]. Moreover, compared to performing two separate tests, the dual-component co-detection of CA199 and AFP saved time and resources, facilitating the diagnosis of gastrointestinal tumors and enabling a more comprehensive assessment of patients’ health status. And it can simultaneously identify pancreatic cancer via elevated CA199 levels and liver cancer via increased AFP levels, thereby facilitating early disease diagnosis [26,38]. The results indicated that the DMSNs-BCDs-based LFIA can rapidly, sensitively, and quantitatively detect PCT, AFP, and CA199, holding great promise for the immediate detection of various markers [39,40].

## 2. Materials and Methods

### 2.1. Reagents

Triethanolamine, cetyltrimethyl ammonium bromide (CTAB), sodium salicylate, tetraethyl silicate (TEOS), ethanol, concentrated hydrochloric acid (HCl), ammonia, β-(aminoethyl)-γ-aminopropyltrimethoxysilane (AEAPTMS), γ-aminopropyltriethoxysilane (APTES) citric acid (CA), sodium dihydrogen phosphate (MSP), absolute ethanol, N,N-Dimethylformamide (DMF), Tween-20, Polyvinyl Pyrrolidone (PVP), NaCl, and succinic anhydride were purchased from Sinopharm Chemical Reagent Co., Ltd. (Shanghai, China). 1-(3-Dimethylaminopropyl)-3-ethylcarbodiimide hydrochloride (EDC) was purchased from Energy Chemical Co., Ltd. (Tangshan, China). Bovine serum albumin (BSA) was purchased from Sangon Biotech Co., Ltd. (Shanghai, China). The procalcitonin antigen (PCT) and its matched labeled antibody and coated antibody were provided by Nanjing Okai Biotechnology Co., Ltd. (Nanjing, China). The AFP, CA199, and its matched labeled antibody and coated antibody were provided by the Jiangsu Provincial Center for Disease Control and Prevention. Goat anti-mouse immunoglobulin G (IgG), sample pad, nitrocellulose (NC) membrane, absorbent pad, and polyvinyl chloride (PVC) substrate were purchased from Shanghai Jieyi Biotechnology Co., Ltd. (Shanghai, China). 

### 2.2. Synthesis of DMSNs-BCDs

DMSNs were prepared according to previous reports [41]. Then, 10 mg of DMSNs was dispersed in 10 mL of ethanol and mixed with 36 μL of AEAPTMS. After homogenization, 0.2 mL of ammonia was added. The mixture was stirred for 12 h, after which the precipitates were separated and washed to obtain AEAPTMS-grafted silica nanospheres (DMSNs-AEAPTMS). The DMSNs-AEAPTMS were dispersed in 7 mL of DMF, which was followed by the addition of 1 mL of CA (4 mg/mL) and 1 mL of MSP (5 mg/mL). The mixture was well mixed and transferred to a Teflon-lined autoclave. The reaction was carried out in an oven at 220 °C for 45 min. After natural cooling, the product was centrifuged and washed with ethanol three times. Next, 10 mg of DMSNs-BCDs were dispersed in 10 mL of ethanol, which was followed by the addition of 40 μL of APTES and 200 μL of ammonia. The mixture was stirred for 6 h, centrifuged, washed, and dispersed in 10 mL of DMF. Subsequently, 100 mg of succinic anhydride was added, and the mixture was stirred for 4 h. The product (DMSNs-BCDs-NH_2_) was obtained by centrifugation and washing to yield carboxyl-terminated DMSNs-BCDs (DMSNs-BCDs-COOH).

### 2.3. Measurement of Fluorescence Quantum Yield

Fluorescence quantum yield was determined using the reference method with the aid of an RF-6000 fluorescence spectrophotometer. A 0.05 mol/L H_2_SO_4_ solution of quinine sulfite is a quantum yield reference material that has a quantum yield of 0.54 under excitation at a wavelength of 360 nm. The specific process is as follows: measure the fluorescence emission spectrum of the dilute solution of the sample to be measured and the dilute solution of the reference fluorescent substance under the excitation of 360 nm and integrate the area of the emission spectrum peak. At the same time, measure the absorbance at 360 nm, and then the fluorescence quantum yield of the sample to be measured can be calculated according to the following formula.

(1) Φu=ΦsFuAs/FsAu

In the formula, *Φu* and *Φs* represent the fluorescence quantum yields of the test substance and the reference substance, respectively; *Fu* and *Fs* represent the fluorescence integral area of the test substance and the reference substance, respectively; and *Au* and *As* represent the absorbance of the test substance and the reference substance, respectively [42].

### 2.4. Preparation of Sample (DMSNs-BCDs Label Antibody)

To prepare the sample, 250 μL of DMSNs-BCDs-COOH (1 mg/mL), 20 μL of EDC aqueous solution (10 mg/mL), and 10 μL of anti-PCT-Ab_2_ (R151h2, 5 mg/mL) were sequentially mixed and gently shaken for 2 h at room temperature. The conjugates were centrifuged and dispersed in 250 μL of 0.01 M PBS solution and stored at 4 °C. The sample pads were treated with a mixed solution (containing 0.05% Tween-20, 0.1% PVP, 0.5% BSA, and 2% NaCl) and then dried at room temperature. The test line and control line on the nitrocellulose (NC) membrane were coated with the corresponding capture antibodies (R150e6, 2 mg/mL) and goat anti-mouse IgG antibodies (2 mg/mL), respectively. Subsequently, the absorbent pad, NC membrane, and pre-treated sample pad were affixed onto a PVC backing card, cut into strips of 4 mm width, and then stored in the dark at 4 °C. Next, 5 μL of DMSNs-BCDs-labeled antibody solution was mixed with 35 μL of PCT standard solution at different concentrations and then applied to the sample pad.

Measures of 250 μL of DMSNs-BCDs-COOH (1 mg/mL), 20 μL of EDC aqueous solution (10 mg/mL), and 10 μL of anti-AFP (Ab_2_, 7.5 mg/mL) were sequentially mixed and gently shaken for 2 h at room temperature. Likewise, 250 μL of DMSNs-BCDs-COOH (1 mg/mL), 20 μL of EDC aqueous solution (10 mg/mL), and 10 μL of anti-CA199 (Ab_2_, 6.5 mg/mL) were sequentially mixed and gently shaken for 2 h at room temperature. The sample pads were processed following the same procedure as described above. Anti-AFP (Ab_1_, 2 mg/mL), anti-CA199 (Ab_1_, 2 mg/mL), and goat anti-mouse IgG antibody (2 mg/mL) were dispensed onto the T_1_, T_2_, and C lines on the NC membrane using a pen and then allowed to dry at room temperature. Subsequent procedures were performed following the same protocol as mentioned above. Measures of 5 μL of DMSNs-BCDs-Ab_2_(AFP/CA199) were mixed with 30 μL of AFP-Ag solution and 30 μL of CA199-Ag solution at different concentrations, respectively. The two mixtures were then combined and applied to the sample pads.

### 2.5. Detection of DMSNs-BCDs-Based LFIA

The prepared samples were applied onto the sample pad of the LFIA, and the results were read after a 15 min incubation period. Fluorescence images of the test strips were captured using a Huawei phone under a 365 nm ultraviolet lamp, and ImageJ software (ImageJ 1.53t) was utilized for analyzing the fluorescence intensity of both the control and test lines. Three replicate measurements were carried out for each concentration [43].

## 3. Results and Discussion

### 3.1. Synthesis and Characterization of DMSNs-BCDs

**Figure 2:**
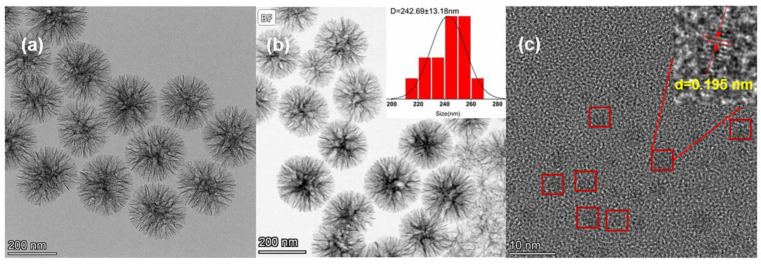
(**a**) TEM images of DMSNs; (**b**) TEM image of DMSNs-BCDs and the corresponding particle size distribution (inset); (**c**) TEM image of the supernatant obtained after centrifugation following the hydrothermal reaction and the lattice fringe image (inset).

The chemical synthesis route of the DMSNs-BCDs is shown in Figure 1a. CA and MSP were used as precursors, DMSNs-AEAPTMS as the carrier, and DMF as the solvent. BCDs can be easily grown *in situ* within DMSNs spheres using the hydrothermal method. These two steps are characterized by simplicity and high yield, leading to the synthesis of DMSNs-BCDs with strong fluorescence, which is advantageous for their application in LFIA test strips. To confirm the structure and elemental composition of the DMSNs and DMSNs-BCDs, transmission electron microscopy (TEM) was employed to analyze the micro-morphology; it can be observed from Figure 2a,b that there is minimal impact on the visual appearance and pore structure before and after load. TEM images demonstrate that DMSNs-BCDs exhibit good dispersion with particle sizes ranging from 230 to 260 nm. Figure 2c displays the TEM image of the supernatant obtained after centrifugation following the hydrothermal reaction, revealing the well-defined lattice patterns of carbon dots. The calculated lattice spacing of the carbon dots is 0.195 nm, providing further evidence for the successful synthesis of BCDs [44]. BCD precursors are in excess, resulting in the excessive synthesis of carbon dots. However, the quantity of BCDs loaded onto the DMSNs is limited. As a result, residual carbon dots are present in the supernatant. The EDS elemental mapping indicates that DMSNs-BCDs are composed of Si, O, C, and P elements (Figure S1), demonstrating the successful doping of phosphorus.

**Figure 3:**
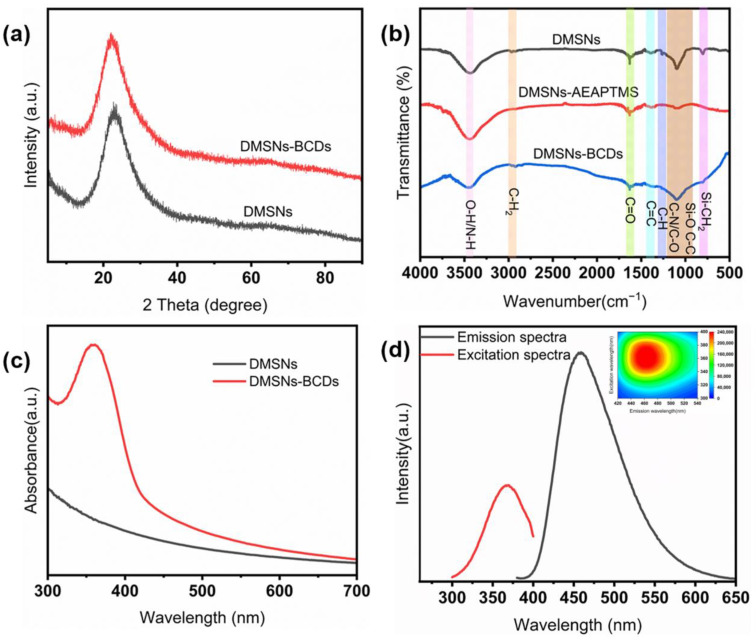
(**a**) XRD of DMSNs and DMSNs-BCDs; (**b**) FT-IR spectra of DMSNs and DMSNs-BCDs; (**c**) UV-vis absorption spectra of DMSNs and DMSNs-BCDs; and (**d**) fluorescence emission spectra and excitation spectra of DMSNs-BCDs, along with the 3D fluorescence of DMSNs-BCDs in aqueous solution (insert).

The composition of the DMSNs-BCDs was studied using X-ray diffraction (XRD), Fourier transform infrared (FT-IR) technology, UV-visible absorption, and fluorescence spectra. The XRD diagram is shown in Figure 3a. The DMSNs and DMSNs-BCDs show a wide peak centered at 22°, indicating that CDs grow on the surface of DMSNs without fixed carbon. The functional groups of DMSNs-BCDs were studied in depth using Fourier transform infrared spectroscopy (FT-IR) (Figure 3b). The characteristics of DMSNs are basically the same as reported in the literature [36]. For DMSNs-BCDs, these include O-H (3450 cm^−1^), C-H_2_ (2918, 2850 cm^−1^), C-O (1657 cm^−1^), C-C (1564 cm^−1^), C-H (1383 cm^−1^), and Si-CH_2_ (806 cm^−1^). Additionally, the strong and wide absorption band at 900–1300 cm^−1^ belongs to C-C, Si-O, and C-O/C-N. The enhanced absorption peak intensity at 3450 cm^−1^ for DMSNs-AEAPTMS indicates the presence of N-H, which further confirms the reaction mechanism illustrated in Figure 1a. The entire reaction mechanism can be described as follows: The surfaces of the DMSNs contain a large amount of -OH, while AEAPTMS is an alkylating agent with an alkoxy group at one end and an amino group at the other. During the grafting of AEAPTMS onto the DMSNs, ammonia promotes the hydrolysis of the alkylating agent, forming Si-O-Si bonds. The amino group at the tail end of AEAPTMS can directly bond with CA, and MSP is doped in the form of a phosphorus source during the bonding process. As shown in Figure 3c, the absorption peak of DMSNs-BCDs occurs at approximately 360 nm. This further proves the successful *in situ* synthesis of carbon dots. As shown in Figure 3d, the emission wavelength of DMSNs-BCDs is 460 nm under the optimal excitation wavelength of 360 nm. DMSNs themselves are non-fluorescent; it is the successful synthesis of carbon dots that imparts fluorescence. At the optimal excitation wavelength of 360 nm, the emission wavelength of DMSN-BCDs is 460 nm. This is corroborated by the 3D fluorescence spectrum, further affirming the significance of DMSNs as carriers. The horizontal axis represents emission wavelength, while the vertical axis represents excitation wavelength (Figure S2). 

Due to the successful preparation of DMSNs-BCDs, which is the first-ever composite fluorescent nanosphere, it is necessary to explore the concentration and type of precursors, reaction temperature, reaction time, acid and alkali resistance, and storage time of the material in order to obtain high-quality DMSNs-BCDs. The DMSNs-AEAPTMS were subjected to hydrothermal reactions with aqueous solutions of CA and a solution containing CA and MSP, as shown in Figure S3. The group containing both CA and MSP exhibited the highest fluorescence intensity. Furthermore, the DMSNs-AEAPTMS were dispersed in a mixture containing 7 mL of DMF and 2 mL of various aqueous solutions containing varying amounts of CA and MSP, at mass ratios of CA to MSP of 4:2.5, 4:5, 4:6, 3.5:5, and 6:5. This resulted in the preparation of DMSNs-BCDs. The reaction was carried out in a high-pressure reactor at 220 °C for 45 min. As shown in Figure S4, the comparison of fluorescence intensity revealed that the highest fluorescence intensity was achieved when the mass ratio of CA to MSP in the aqueous solution was 4:5. The incorporation of phosphorus as a dopant is crucial for enhancing the fluorescence of BCDs. The doping of phosphorus introduces new energy levels, which interact with the energy level structure of carbon dots, promoting electron transitions and thereby enhancing fluorescence emission. Phosphorus doping can reduce the non-radiative recombination process, increase the fluorescence quantum yield, and enable carbon dots to emit light more effectively. It can also improve the fluorescence stability of carbon dots, reduce the decay rate of the fluorescence signal, and thus prolong the duration of the fluorescence signal [32,45,46,47,48]. This is one of the reasons for the high fluorescence quantum yield of DMSNs-BCDs. Subsequently, we conducted further experiments at different temperatures and with different durations, including 220 °C (0.5 h, 45 min, 1 h), 200 °C (0.5 h, 1 h), and 180 °C (0.5 h, 1 h, 2 h, 8 h, 10 h), as shown in Figure S5. The experimental results indicated that the highest fluorescence intensity and emission peak at 460 nm were achieved when the reaction temperature was set at 220 °C and the reaction time lasted for 45 minutes. For the safety of the experiment, the temperature was not increased. To further explore the acid-base resistance of the DMSNs-BCDs material, we evaluated the stability of its signal intensity at different pH values (ranging from 1 to 13). As depicted in Figure S6, DMSNs-BCDs demonstrated good stability in acidic and alkaline solutions. In addition, the signal intensity stability of DMSNs-BCDs under long-term storage conditions was evaluated. The results showed that the fluorescence intensity of DMSNs-BCDs is almost unaffected by time, meeting the requirements of LFIA (Figure S7).

The aforementioned characteristic results indicate the successful preparation of DMSNs-BCDs. These possess advantages such as simple synthesis, good dispersibility, high quantum yield, and fluorescence stability, meeting the requirements for LFIA.

### 3.2. Performance of DMSNs-BCDs-LFIA for PCT, CA199, and AFP Detection

Based on the excellent fluorescence characteristics, stability, and dispersibility of DMSNs-BCDs, we utilized them as fluorescent signal molecules for the detection of PCT, CA199, and AFP as target analytes in an LFIA format. As depicted in Figure 1b,c, following the successful conjugation of detection antibodies onto the surface of DMSNs-BCDs, the test strip was further modified for the specific detection of PCT and tumor biomarkers. The NC membrane was functionalized with capture antibodies and IgG. Subsequently, the sample was applied to the sample pad, allowing it to migrate along the test strip through capillary action. In the presence of the target analytes (PCT/CA199/AFP), the DMSNs-BCDs-antibody conjugates were selectively captured at the test line via sandwich immune reactions, resulting in a positive fluorescence signal. Conversely, the lack of DMSNs-BCDs fluorescence at the test line indicating the absence of the target antigens in the sample, yielded a negative result. In both scenarios (presence and absence of the antigen), the DMSNs-BCDs–antibody conjugates bound to the control line through IgG binding. The visualization of the test strip was conducted using a 365 nm ultraviolet lamp. The quantitative concentration of the analyte in the sample was further determined by calculating the ratio between the fluorescence intensity of the test line and the control line (T/C).

**Figure 4:**
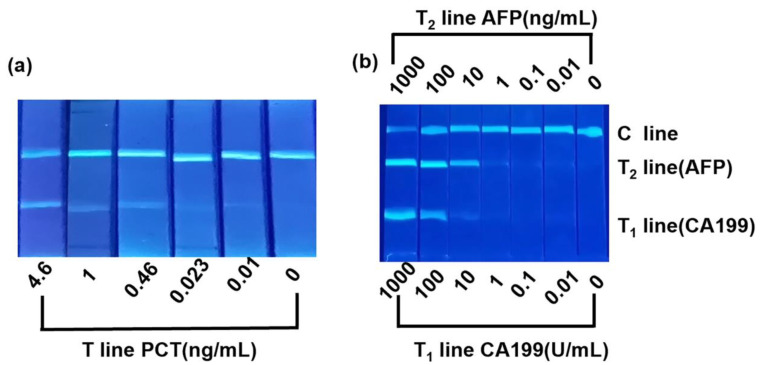
(**a**) Picture of the DMSNs-BCDs-LFIA detecting different concentrations of PCT solutions; (**b**) DMSNs-BCDs-LFIA two-component detection photographs of CA199 and AFP.

**Figure 5:**
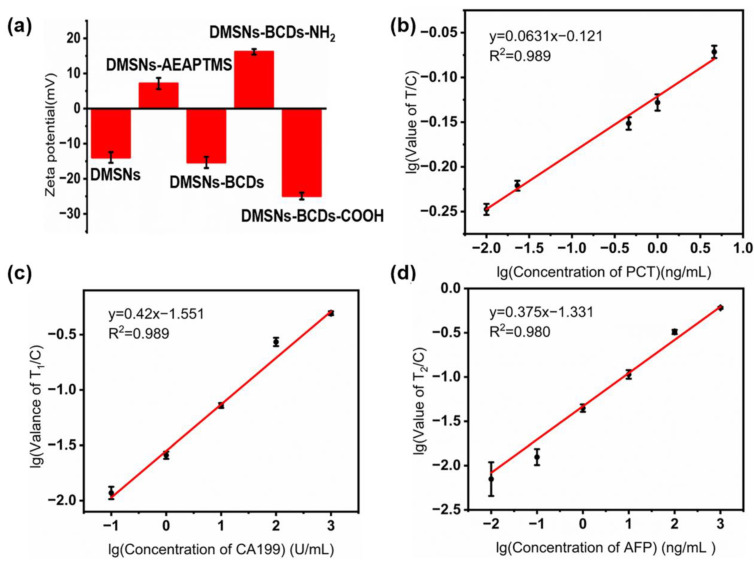
(**a**) ζ potential of DMSNs, DMSNs-AEAPTMS, DMSNs-BCDs, DMSNs-BCDs-NH_2_, and DMSNs-BCDs-COOH; (**b**) linear response of the DMSNs-BCDs-LFIA to PCT detection, with a concentration range of 4.6 ng/mL–0.01 ng/mL; (**c**) linear response of the DMSNs-BCDs-LFIA to CA199 detection, with a concentration range of 1000 U/mL–0.1 U/mL; and (**d**) linear response of the DMSNs-BCDs-LFIA to AFP detection, with a concentration range of 1000 ng/mL–0.01 ng/mL.

PCT antigen standard solutions at different concentrations (0, 0.01, 0.023, 0.46, 1, 4.6 ng/mL) were used to analyze the sensitivity of the DMSNs-BCDs-LFIA. The test strip results were imaged using a Huawei mobile phone, as shown in Figure 4a. All control lines on the test strips displayed fluorescent signals, indicating that the test results were valid. The results showed that the LOD for PCT was approximately 0.01 ng/mL. The DMSNs-BCDs-LFIA fully leveraged the high fluorescence intensity of BCDs to successfully lower the detection limit. The signal intensities of the test and control lines were quantified using ImageJ software. Throughout the preparation process, the potential results for DMSNs, DMSNs-AEAPTMS, DMSNs-BCDs, DMSNs-BCDs-NH_2_, and DMSNs-BCDs-COOH are shown in Figure 5a. Compared to DMSNs, the potential of DMSNs-AEAPTMS changed from negative (ζ potential = −13.93 mV) to positive (ζ potential = 7.14 mV), which may be due to the grafting of AEAPTMS onto DMSNs with hydroxyl-covered surfaces. After the *in situ* growth of CDs, the surfaces of DMSNs-BCDs contain O-H and C=O, which exhibit electronegativity (ζ potential = −15.34 mV). Subsequently, it was aminated and became positively charged (ζ potential = 16.16 mV). Afterwards, the amine groups were carboxylated with succinic anhydride, and the composite sphere surface was filled with carboxyl groups, exhibiting electronegativity (ζ potential = −24.92 mV). The results indicate that the reactions proceeded smoothly at each stage. The relationship between brightness of the test line and the concentration of PCT analyzed by ImageJ software is y = 0.0631x − 0.121 (R^2^ = 0.989), as shown in Figure 5b.

CA199 and AFP antigen standard solutions at different concentrations (CA199: 0, 0.01, 0.1, 1, 10, 100, and 1000 U/mL; AFP: 0, 0.01, 0.1, 10, 100, and 1000 ng/mL) were used to analyze the sensitivity of DMSNs-BCDs-LFIA. As shown in Figure 4b, all control lines on the test strips displayed fluorescent signals, indicating that the test results were valid. The results showed that the LOD for CA199 was approximately 1 U/mL and for AFP was approximately 0.01 ng/mL. The signal strength of the test and control lines was obtained using ImageJ software. As shown in Figure 5c,d, a linear relationship was observed between the T-line intensity value/C-line intensity value for CA199 and AFP and the antigen concentration, where y = 0.420x − 1.551 (R^2^ = 0.989) and y = 0.375x − 1.331 (R^2^ = 0.980), respectively.

### 3.3. Stability and Specificity Detection of DMSNs-BCDs-LFIA

The capability to assess intra-assay and inter-assay coefficient of variation (CV) and to accurately identify target analytes are essential characteristics of DMSNs-BCDs-LFIA. The precision of DMSNs-BCDs-LFIA was evaluated using a standard solution containing 0.05 ng/mL of PCT antigen. Additionally, the precision of DMSNs-BCDs-LFIA was assessed using standard solutions of CA199 antigen at concentrations of 0.05 U/mL and 0.1 U/mL, as well as AFP antigen standard solutions at concentrations of 0.05 ng/mL and 0.1 ng/mL. The mean (*M*) and standard deviation (*SD*) were calculated, and the *CV* was determined as follows [49]:

(2) CV=(SD/M) %

**Figure 6:**
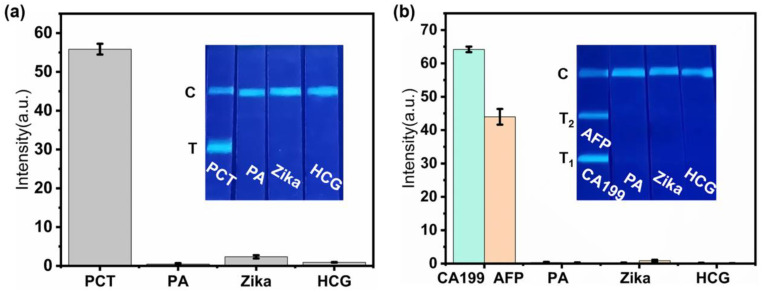
Study on the specificity of a DMSNs-BCDs-LFIA test strip to different interfering proteins. ((**a**) single-component detection; (**b**) dual-component simultaneous detection).

Intra-assay and inter-assay measurements were conducted in quintuplicate. As presented in Table S1, both the intra-assay and inter-assay coefficients of variation (CVs) were found to be below 11%, indicating that the DMSNs-BCDs-LFIA exhibits highly satisfactory accuracy. Moreover, the specificity of DMSNs-BCDs-LFIA was validated by assessing three biomarkers namely, PA, Zika, and HCG at a concentration of 1 μg/mL. As illustrated in Figure 6a, with the exception of PCT, no blue fluorescence was observed on the test line for the other three biomarkers, thus demonstrating the excellent selectivity of DMSNs-BCDs-LFIA. Additionally, Table S3 provides a comparative analysis of the analytical performance of various methods for PCT detection.

As depicted in Table S2, the intra-assay and inter-assay CVs for CA199 were found to be less than 10%, while the intra-assay and inter-assay CVs for AFP were less than 11%, indicating the excellent accuracy of DMSNs-BCDs-LFIA. Moreover, the specificity of DMSNs-BCDs-LFIA was validated by assessing three biomarkers namely, PA, Zika, and HCG at a concentration of 1 μg/mL. As demonstrated in Figure 6b, except for CA199 and AFP, no blue fluorescence was observed on the test line for the other three biomarkers, highlighting the robust selectivity of DMSNs-BCDs-LFIA. Furthermore, Table S4 presents a comparative analysis of the analytical performance of diverse methods for CA199 and AFP detection. DMSNs-BCDs-LFIA offers the advantages of rapidity and convenience. When compared to fluorescence sensing methods, DMSNs-BCDs-LFIA exhibits a wider linear range and lower LOD, thus showcasing superior analytical performance.

## 4. Conclusions

In summary, we have overcome the challenges involved in the connection between signal molecules and carriers and have successfully developed a rapidly responsive, selective, and effective LFIA utilizing high-quantum-yield, fluorescent nanomaterials (DMSNs-BCDs). The issue of low sensitivity in LFIA due to the low fluorescence intensity of signal molecules has been addressed and resolved. By leveraging the ultra-high-brightness nanomaterials in LFIA, we achieved the rapid and specific detection of PCT as well as CA199/AFP within 15 min and sharply decreased the LOD compared with conventional colloidal gold strips. This highly fluorescent nanomaterial is expected to be applied in the preparation of novel label materials to further improve the performance of LFIA. Furthermore, DMSNs-BCDs-LFIA is portable, cost-effective, and holds significant implications for practical sample analysis and clinical diagnostics.

## Data Availability

Data is contained within the article or Supplementary Material.

## Supplementary Materials

The following supporting information can be downloaded at: https://www.mdpi.com/article/10.3390/s24010019/s1, Figure S1: EDS spectrum DMSNs-BCDs; Figure S2: Photos of the DMSNs-BCDs solids under daylight and UV light; Figure S3: Effect of the type of precursor on the fluorescence performance of DMSNs-BCDs; Figure S4: The effect of CA and MSP concentration on the fluorescence properties of DMSNs-BCDs; Figure S5: The effect of reaction time and temperature on the fluorescence performance of DMSNs-BCDs; Figure S6: Photos of DMSNs-BCDs aqueous solutions at different pH values under a 365 nm fluorescent lamp; Figure S7: Fluorescence signal intensity of DMSNs-BCDs under long-term storage; Tables S1 and S2: The results of the coefficient of variation (CV) of DMSNs-BCDs-LFIA (PCT, CA199, and AFP); Tables S3 and S4: Summary of PCT, CA199, and AFP detection with some different detection methods.
