# Supplementary material for: In Situ Synthesis of Highly Fluorescent, Phosphorus-Doping Carbon-Dot-Functionalized, Dendritic Silica Nanoparticles Applied for Multi-Component Lateral Flow Immunoassay
Source: Sensors (Basel). 2023 Dec 19;24(1):19. doi: 10.3390/s24010019 (PMC10780618; doi:10.3390/s24010019)
Supplement: Supplementary file 1 [file sensors-24-00019-s001.zip › sensors-2764133-supplementary.pdf]

Supplementary Materials for

In-Situ Synthesis Highly Fluorescent Phosphorus-Doping Carbon Dots-Functionalized Dendritic Silica Nanoparticles Applied for Multi-Component Lateral Flow Immunoassay

Jia-Xuan Hu and Shou-Nian Ding *

School of Chemistry and Chemical Engineering, Southeast University, Nanjing 211189, China

* Correspondence: snding@seu.edu.cn; Fax: (+86) 25-52090621

Characterization

UV-vis absorbance spectra were obtained on a Shimadzu UV 2600 spectropolarimeter (Japan). Transmission electron microscopy (TEM) measurements were carried out under a field-emission high-resolution transmission electron microscopy Talos F200X (Thermo Scientific, USA). Fluorescence spectra, three-dimensional (3D) fluorescence spectra and relative quantum yields were performed on a Shimadzu RF-6000 Spectro Fluorophotometer (Japan). Fourier transform infrared (FT-IR) spectrum was collected from a Nicolet 5700 (Thermo Nicolet Corporation, USA) IR spectrometer in the range of 4000-500 cm^{-1}. Powder X-ray diffraction (XRD) dates were measured using a Bruker D8 discover X-ray diffractometer (Bruker, Germany). Zeta (ζ) potential was carried out at 25 °C using a Zetasizer Nano-ZS from Malvern Instruments.

Ethical Statement

All experiments were carried out in accordance with the relevant laws and institutional guidelines as per the Institutional Ethics Committee of Southeast University. The School of Chemistry and Chemical Engineering, Southeast University has approved the experiments.

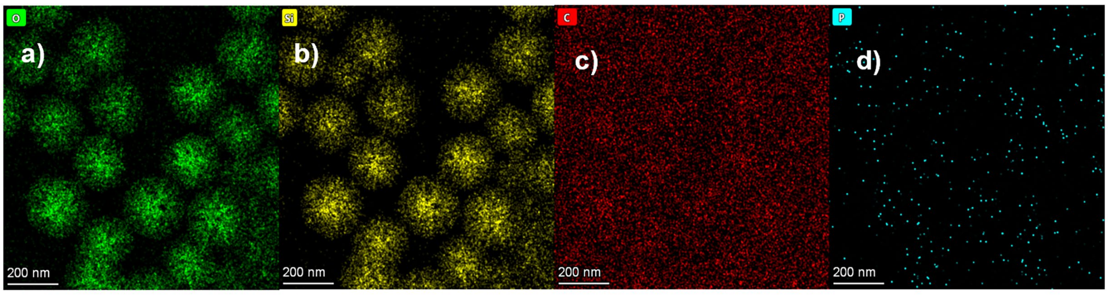

Figure S1. (**a**–**d**) EDS mapping images of DMSNs-BCDs.

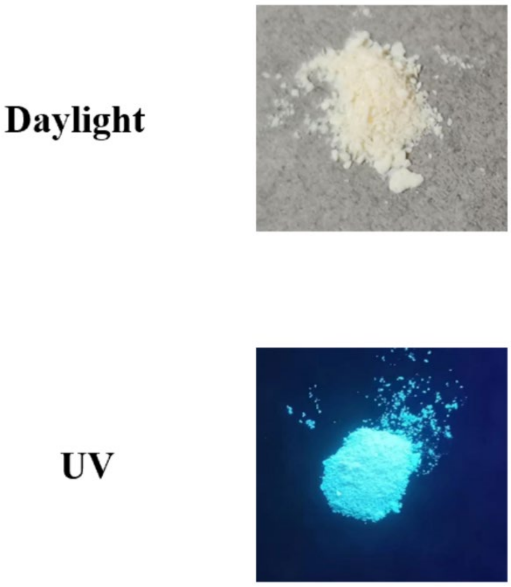

Figure S2. Photos of the DMSNs-BCDs solids under daylight and UV light.

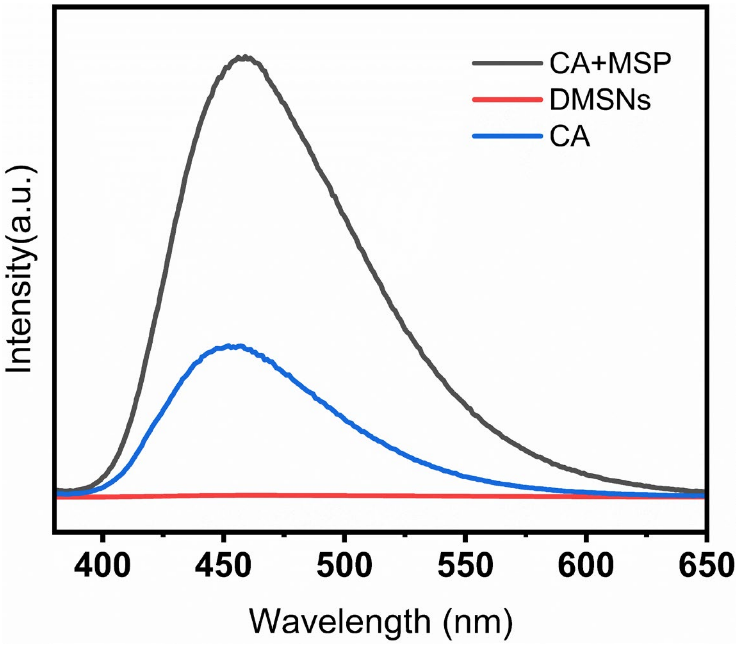

Figure S3. Effect of the type of precursor on the fluorescence performance of DMSNs-BCDs.

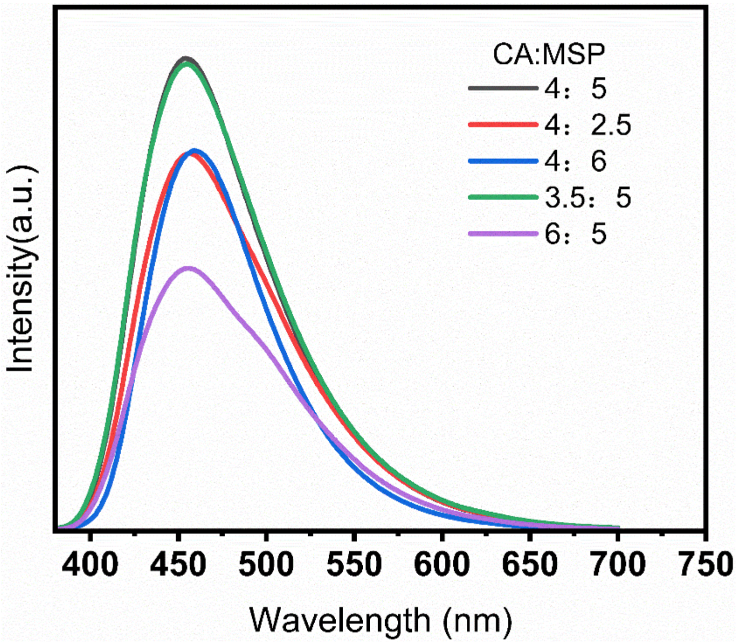

Figure S4. The effect of CA and MSP concentration on the fluorescence properties of DMSNs-BCDs.

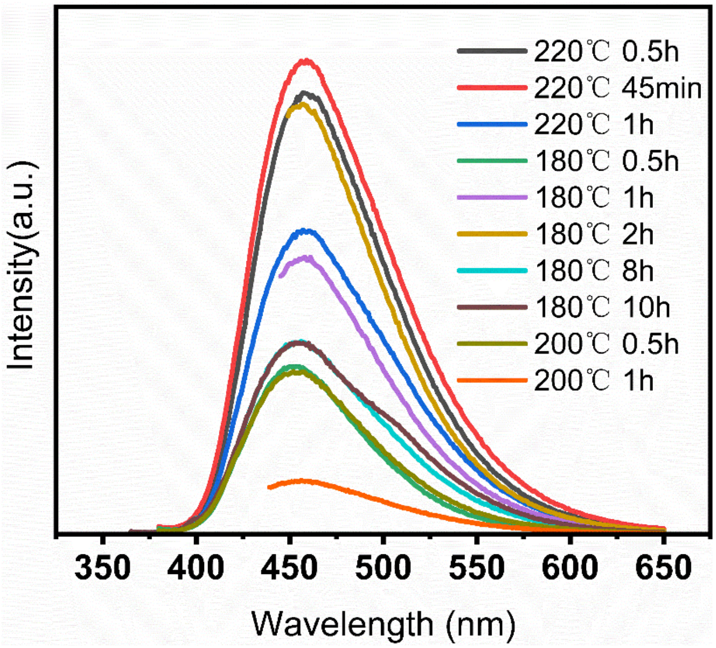

Figure S5. The effect of reaction time and temperature on the fluorescence performance of DMSNs-BCDs.

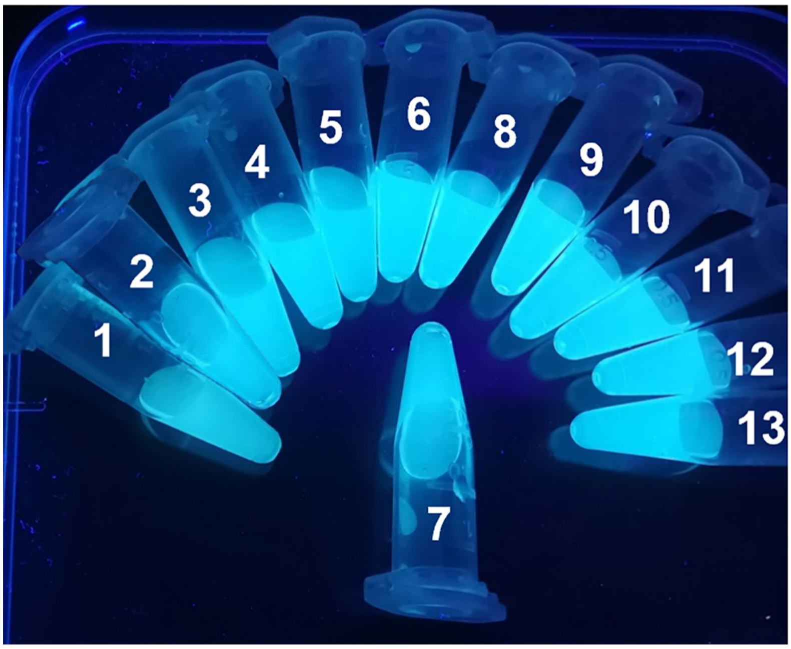

Figure S6. Photos of DMSNs-BCDs aqueous solutions at different pH values under a 365nm fluorescent lamp.

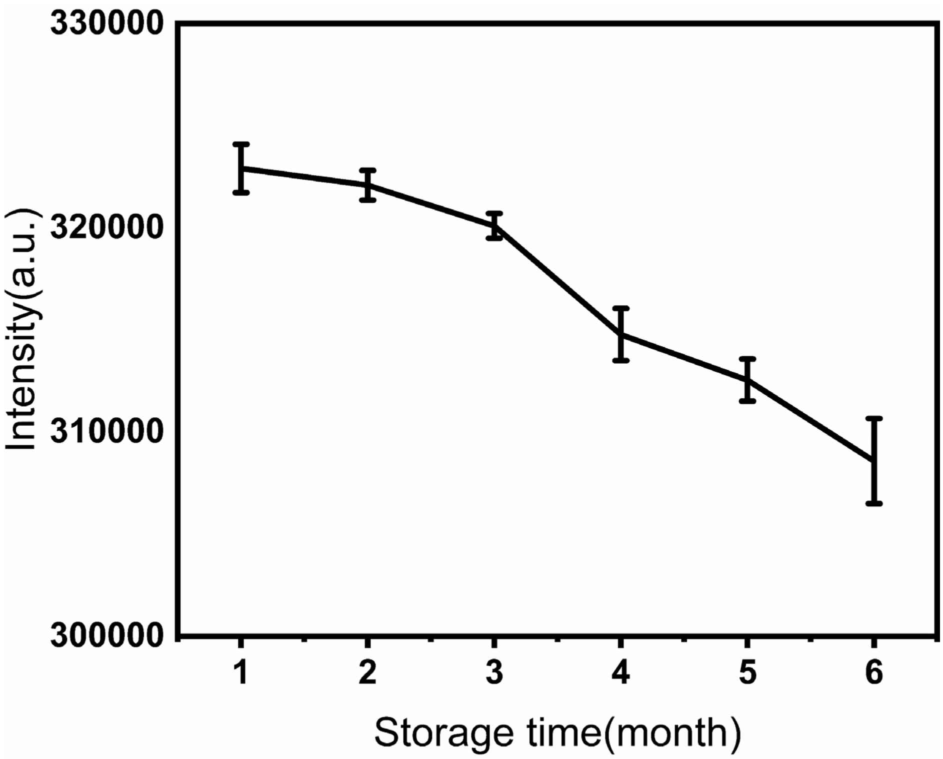

Figure S7. Fluorescence signal intensity of DMSNs-BCDs under long-term storage.

Table S1. The results of the coefficient of variation (CV) of DMSNs-BCDs-LFIA (PCT).

Sample (ng/mL)	Intra-CV (%)	Inter-CV (%)
0.05	8.14±0.24	10.3±0.41

Table S2. The results of the coefficient of variation (CV) of DMSNs-BCDs-LFIA (CA199 and AFP).

CA199			AFP		
Sample (U/mL)	Intra-CV (%)	Inter-CV (%)	Sample (ng/mL)	Intra-CV (%)	Inter-CV (%)
50	6.32±0.15	8.54±0.28	50	9.68±0.71	10.13±0.73
100	7.75±0.22	8.49±0.63	100	5.04±0.87	7.59±0.51

Table S3. Summary of PCT detection with some different detection methods.

Methods	Limit of Detections	Detection time	Disadvantage
Electrochemiluminescence immunoassay	0.17 pg/mL[1]	18 min	Not portable
chemiluminescence immunoassay	7.5 pg/mL[2]	30 min	Short luminescence process; Poor stability
Lateral Flow Immunoassay-Au	0.5 ng/mL[3]	15 min	Low sensitivity
Lateral Flow Immunoassay-QDs	0.0625 ng/mL[4]	15 min	Low sensitivity
this work	0.01 ng/mL	15 min	

Table S4. Summary of CA199 and AFP detection with some different detection methods.

Methods	Limit of Detections	
	CA199 (U/mL)	AFP (ng/mL)
Electrochemical method	0.228[5]	0.005[6]
Chemiluminescence	0.2[7]	6[8]
Lateral Flow Immunoassay-Au	16[9]	1[10]
Fluorescence immunoassay	4.125[11]	0.05[12]
Microchip electrophoresis	0.36[13]	0.35[13]
this work	1	0.01

References

1. Chen, P. P.; Qiao, X. Y.; Liu, J. H.; Xia, F. Q.; Tian, D.; Zhou, C. L. A dual-signals response electrochemiluminescence immunosensor based on PTC-DEPA/KCC-1 NCs for detection of procalcitonin. *Sens. Actuators B Chem.* **2018**, *267*, 525-532. DOI: 10.1016/j.snb.2018.04.061.
2. Wang, G.; Wan, Y.; Lin, G. F.; Li, Z. X.; Dong, Z. N.; Liu, T. C. Development of a novel chemiluminescence immunoassay for the detection of procalcitonin. *J. Immunol. Methods* **2020**, *484*, 4. DOI: 10.1016/j.jim.2020.112829.
3. Serebrennikova, K. V.; Samsonova, J. V.; Osipov, A. P. A semi-quantitative rapid multi-range gradient lateral flow immunoassay for procalcitonin. *Mikrochim Acta* **2019**, *186* (7), 8. DOI: 10.1007/s00604-019-3550-2.
4. Zhou, S.; Peng, Y. L.; Hu, J.; Duan, H.; Ma, T. T.; Hou, L.; Li, X. M.; Xiong, Y. H. Quantum dot nanobead-based immunochromatographic assay for the quantitative detection of the procalcitonin antigen in serum samples. *Microchem. J.* **2020**, 159, 7. DOI: 10.1016/j.microc.2020.105533.
5. Wang, R.; Feng, J. J.; Liu, W. D.; Jiang, L. Y.; Wang, A. J. A novel label-free electrochemical immunosensor based on the enhanced catalytic currents of oxygen reduction by AuAg hollow nanocrystals for detecting carbohydrate antigen 199. *Biosens. Bioelectron.* **2017**, *96*, 152-158. DOI: 10.1016/j.bios.2017.05.007.
6. Sun, J.; Tian, D.; Guo, Q.; Zhang, L.; Jiang, W.; Yang, M. A label-free electrochemical immunosensor for the detection of cancer biomarker α-fetoprotein (AFP) based on hydroxyapatite induced redox current. *Anal. Methods* **2016**, *8* (40), 7319-7323. DOI: 10.1039/c6ay02555b.
7. Chu, W. R.; Chen, Y.; Liu, W.; Zhao, M.; Li, H. F. Paper-based chemiluminescence immunodevice with temporal controls of reagent transport technique. *Sens. Actuators B Chem.* **2017**, *250*, 324-332. DOI: 10.1016/j.snb.2017.04.126.
8. Zong, C.; Wu, J.; Wang, C.; Ju, H. X.; Yan, F. Chemiluminescence imaging immunoassay of

multiple tumor markers for cancer screening. *Anal. Chem.* **2012**, *84* (5), 2410-2415. DOI: 10.1021/ac203179g.

9. Jiao, X.; Peng, T.; Liang, Z.; Hu, Y.; Meng, B.; Zhao, Y.; Xie, J.; Gong, X.; Jiang, Y.; Fang, X.; et al. Lateral flow immunoassay based on time-resolved fluorescence microspheres for rapid and quantitative screening CA199 in human serum. *Int. J. Mol. Sci.* **2022**, *23* (17). DOI: 10.3390/ijms23179991.
10. Lu, L. C.; Yu, J. L.; Liu, X. X.; Yang, X. S.; Zhou, Z. H.; Jin, Q.; Xiao, R.; Wang, C. W. Rapid, quantitative and ultra-sensitive detection of cancer biomarker by a SERRS-based lateral flow immunoassay using bovine serum albumin coated Au nanorods. *RSC Adv.* **2020**, *10* (1), 271-281. DOI: 10.1039/c9ra09471g.
11. Wang, C.W.; Shen, W.Z.; Rong, Z.; Liu, X.X.; Gu, B.; Xiao, R.; Wang, S.Q. Layer-by-layer assembly of magnetic-core dual quantum dot-shell nanocomposites for fluorescence lateral flow detection of bacteria. *Nanoscale* **2020**, *12*, 795–807. https://doi.org/10.1039/c9nr08509b.
12. Jiao, Y.; Du, C.; Zong, L.; Guo, X.; Han, Y.; Zhang, X.; Li, L.; Zhang, C.; Ju, Q.; Liu, J.; et al. 3D vertical-flow paper-based device for simultaneous detection of multiple cancer biomarkers by fluorescent immunoassay. *Sens. Actuators B Chem.* **2020**, 306. DOI: 10.1016/j.snb.2019.127239.
13. Gan, N.; Xie, L. S.; Zhang, K.; Cao, Y. T.; Hu, F. T.; Li, T. H. An endonuclease-linked multiplex immunoassay for tumor markers detection based on microfluidic chip electrophoresis for DNA analysis. *Sens. Actuators B Chem.* **2018**, 272, 526-533. DOI: 10.1016/j.snb.2018.05.071.
